# Effectiveness of Enhanced Monofocal Intraocular Lens with Mini-Monovision in Improving Visual Acuity

**DOI:** 10.3390/jcm14134517

**Published:** 2025-06-26

**Authors:** Santaro Noguchi, Shunsuke Nakakura, Asuka Noguchi, Hitoshi Tabuchi

**Affiliations:** 1 Department of Ophthalmology, Saneikai Tsukazaki Hospital, Himeji 671-1227, Japan; s.nakakura@tsukazaki-eye.net (S.N.); h.tabuchi@tsukazaki-eye.net (H.T.); 2 ASUCA Eye Clinic, Sendai 980-0021, Japan

**Keywords:** enhanced monofocal lens, monovision, cataract surgery, intraocular lens, spectacle dependence

## Abstract

**Objectives**: This study compared the clinical outcomes of Vivinex Impress (XY1-EM) enhanced monofocal and standard Vivinex (XY1) intraocular lenses (IOLs) in mini-monovision cataract surgery. In this retrospective study, patients underwent bilateral implantation with either XY1-EM (33 patients, 66 eyes) or XY1 (24 patients, 48 eyes) in a −1D mini-monovision configuration. **Methods**: Visual acuity was evaluated from 5 m to 30 cm, along with spectacle dependence, contrast sensitivity, and patient-reported outcomes. **Results**: The XY1-EM group demonstrated significantly better intermediate and near visual acuity at distances of 30−50 cm (*p* < 0.05) and reduced spectacle dependence for intermediate distances (*p* = 0.02). Visual function questionnaire (VFQ-25) scores were significantly higher in the XY1-EM group for general vision, role difficulty, mental health, dependency, and near activity domains (*p* < 0.05). No significant differences were found in glare, contrast sensitivity, or quality of vision scores. **Conclusions**: The XY1-EM lens in mini-monovision configuration provides enhanced intermediate and near visual acuity with reduced spectacle dependence compared to standard monofocal IOLs, offering a valuable option for patients seeking improved quality of vision with reduced spectacle use.

## 1. Introduction

Cataract surgery significantly improves visual function and quality of life, but the choice of intraocular lens (IOL) determines the range of functional vision patients experience postoperatively [1]. Traditional monofocal IOLs primarily restore distance vision, leaving patients reliant on spectacles for near and intermediate tasks. While extended depth-of-focus (EDOF) and multifocal IOLs provide broader vision ranges, they often introduce optical side effects and reduced contrast sensitivity [2,3,4,5,6,7,8].

The Vivinex Impress (XY1-EM) enhanced monofocal IOL (Hoya Surgical Optics, Singapore) represents a newer category designed to improve intermediate vision while minimizing visual disturbances. However, the XY1-EM might still have a limited depth of focus, potentially resulting in insufficient near vision and incomplete spectacle independence. The lens design and optical principles of the XY1-EM are entirely undisclosed and remain unknown.

One practical solution to address these limitations is pseudophakic monovision, which extends the functional range of vision by adjusting the refractive targets between eyes. Monovision typically targets emmetropia in the dominant eye and slight myopia in the nondominant eye, improving near and intermediate vision while maintaining distance clarity [9]. Previous studies show that monovision with monofocal IOLs can reduce spectacle dependence with fewer disturbances than multifocal IOLs. Particularly, mini-monovision (−0.75D to −1.75D) effectively balances visual function with minimal side effects [10].

This study aimed to evaluate the clinical outcomes of XY1-EM in a mini-monovision configuration compared with the standard Vivinex monofocal lens (XY1), focusing on visual acuity across distances, spectacle independence, and patient-reported outcomes, including the visual function questionnaire (VFQ-25) and quality of vision (QOV) [11,12]. We hypothesized that the XY1-EM lens can provide superior visual acuity from distant to near and reduce spectacle dependence in patients undergoing mini-monovision cataract surgery.

## 2. Materials and Methods

### 2.1. Study Design

This retrospective observational study evaluated the visual outcomes following mini-monovision cataract surgery using two IOLs: XY1-EM (with an A constant of 119.2) and XY1 (with an A constant of 118.9). The study was conducted in compliance with the principles of the Declaration of Helsinki and was approved by the Ethics Committee of Tsukazaki Hospital (No: 2410009; 15 October 2024). Information about the study was disseminated to patients through hospital notice boards and the website. The necessity for informed consent was waived due to the retrospective nature of the study, with patients having the alternative to discontinue participation.

### 2.2. Participants

Patients who underwent bilateral cataract surgery with a 2.2 mm corneal incision between April 2022 and March 2024 at Tsukazaki Hospital were included. All patients underwent bilateral surgery on the same day by experienced surgeons using the CENTURION phacoemulsification system (Alcon Inc., Fort Worth, TX, USA). In 24 patients (48 eyes), the XY1 was implanted in a mini-monovision configuration. In 33 patients (66 eyes), the XY1-EM was implanted from April 2023 onward.

### 2.3. Surgical Procedure and Targeting

In both groups, the dominant eye was identified preoperatively as the eye preferred when viewing a distant object through a pinhole. Emmetropia was targeted for the dominant eye, while a refractive power closest to −1.0D was targeted for the nondominant eye.

### 2.4. Preoperative and Intraoperative Assessments

Preoperative evaluation included IOL power, axial length, flat and steep keratometry (Kf and Ks), and anterior chamber depth (ACD), which was measured using the IOL Master^®^ 700 (Carl Zeiss, Oberkochen, Germany). IOL power was calculated using the Barrett Universal II formula. Intraoperative cumulative dissipated energy (CDE) was recorded using the phacoemulsification machine. All patients completed the VFQ-25 preoperatively and at 3 months postoperatively. The QOV questionnaire was completed by all patients at 3 months postoperatively.

### 2.5. Postoperative Assessments

At 3 months postoperatively, visual outcomes were assessed, including corrected distance visual acuity (CDVA) and uncorrected visual acuity (UCVA) at 5, 1, 0.7, 0.5, 0.4, and 0.3 m. Visual outcomes were measured using Landolt rings and analyzed using logMAR. Spectacle dependence for far, intermediate, and near distances was surveyed using a three-point scale: “Always used,” “Occasionally,” and “Unused”. Patient-reported outcomes were assessed using the QOV and VFQ-25 questionnaires.

### 2.6. Contrast Sensitivity Evaluation

Contrast sensitivity was measured 3 months after surgery using the CGT-2000 device (Takagi Seiko Co., Ltd., Nagano, Japan). Evaluations included six target sizes (1.1, 1.8, 2.9, 4.5, 7.1, and 10.2 cpd) under mesopic (10 cd/m^2^) and photopic (40,000 cd/m^2^) conditions. The area under the log contrast sensitivity function (AULCSF) was calculated for each condition.

### 2.7. Statistical Analysis

Statistical analysis was performed as follows. Normality was assessed using the Shapiro–Wilk test. Homogeneity of variances was evaluated using the Brown–Forsythe test. For normally distributed data with equal variances, the Student’s *t*-test was used. When variances were unequal, the Welch’s *t*-test (unequal variances *t*-test) was applied. For non-normally distributed data, the Mann–Whitney U test was used. For age and IOL power, due to inconsistent results in the homogeneity tests, the Welch’s *t*-test was applied. The Student’s *t*-test was used for Kf and Ks, whereas the Mann–Whitney U test was used for all other variables. Power analysis was conducted using binocular visual acuity at 0.4 m (logMAR). With α = 0.05, power = 80%, and group sample sizes of 33 (SD = 0.18) and 24 (SD = 0.15), the minimum detectable effect size was 0.13 logMAR. Variables such as sex distribution, spectacle use, and refractive cylinder were analyzed using the chi-square test. Other continuous variables were analyzed using the Mann–Whitney U test. Statistical analyses were performed using SPSS version 26.0 (IBM Corp., Armonk, NY, USA). A *p*-value of <0.05 was considered statistically significant.

## 3. Results

### 3.1. Baseline Characteristics

Table 1 shows the baseline characteristics of the study participants. No statistically significant differences were found in sex, IOL power, axial length, ACD, flat keratometry (Kf), CDE, or CDVA between the XY1 and XY1-EM groups (all *p* > 0.05). The XY1 group had significantly lower steep keratometry (Ks) values than the XY1-EM group (*p* = 0.02). The predicted postoperative refractive values calculated using the Barrett Universal II formula were comparable between groups: −0.18 ± 0.13D vs. −0.16 ± 0.11D for the XY1 and XY1-EM emmetropia groups (*p* = 0.25) and −1.00 ± 0.09D vs. −0.98 ± 0.10D for the XY1 -1D and XY1-EM -1D groups (*p* = 0.72).

### 3.2. Postoperative Visual Acuity

**Figure 1:**
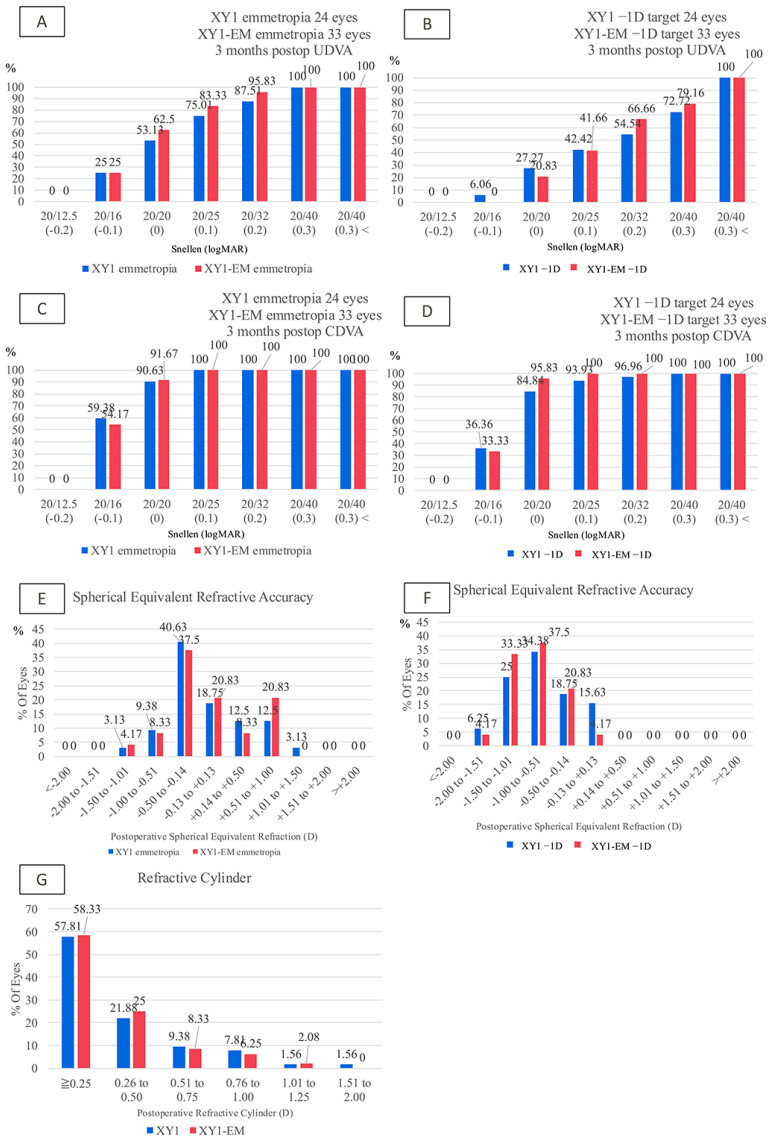
Postoperative visual acuity and refractive outcomes in the dominant and nondominant eyes. (**A**,**B**) Cumulative uncorrected visual acuity (UCVA) and corrected distance visual acuity (CDVA) in emmetropia groups. (**C**,**D**) UCVA and CDVA in −1D groups. (**E**,**F**) Postoperative spherical equivalent refractive outcomes. (**G**) Subjective postoperative astigmatism.

The cumulative uncorrected visual acuity (UCVA) and CDVA showed no significant differences between the XY1 and XY1-EM emmetropia groups (*p* = 0.77, *p* = 0.11) or the XY1 -1D and XY1-EM -1D groups (*p* = 0.79, *p* = 0.40) (Figure 1A–D). Spherical equivalent refractive accuracy and postoperative subjective astigmatism were also comparable between groups (Figure 1E–G).

**Figure 2:**
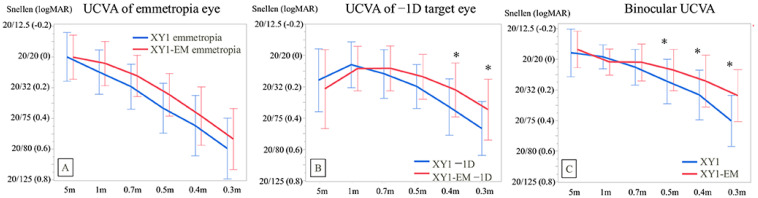
Postoperative visual acuity across various distances. (**A**) UCVA in the dominant eye. (**B**) UCVA in the nondominant eye. (**C**) Binocular UCVA. * *p* < 0.05.

In the dominant eye, the UCVA for XY1 emmetropia vs. XY1-EM emmetropia groups showed no significant differences at any measured distance (all *p* > 0.05) (Figure 2A). However, in the nondominant eye, the XY1-EM −1D group demonstrated significantly better UCVA at 40 cm (0.33 ± 0.19 vs. 0.19 ± 0.17; *p* = 0.01) and 30 cm (0.47 ± 0.19 vs. 0.34 ± 0.23; *p* = 0.03) compared to the XY1 −1D group (Figure 2B).

Most importantly, binocular UCVA in the XY1-EM group was significantly better at 50 cm (0.14 ± 0.14 vs. 0.07 ± 0.14; *p* = 0.01), 40 cm (0.23 ± 0.16 vs. 0.14 ± 0.17; *p* = 0.01), and 30 cm (0.40 ± 0.17 vs. 0.24 ± 0.17; *p* < 0.001) compared to the XY1 group (Figure 2C).

### 3.3. Spectacle Dependence

**Figure 3:**
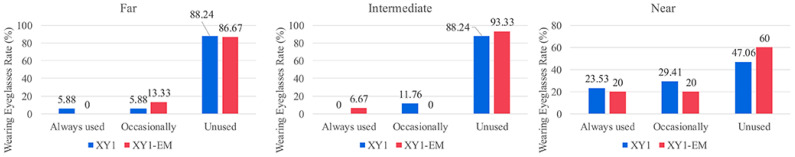
Spectacle dependence across different distances in the study groups.

Spectacle dependence for intermediate distances was significantly lower in the XY1-EM group compared to the XY1 group (*p* = 0.02). However, no significant differences were observed in spectacle use for distance or near vision between the groups (*p* = 0.17 and *p* = 0.56, respectively) (Figure 3).

### 3.4. Contrast Sensitivity

**Figure 4:**
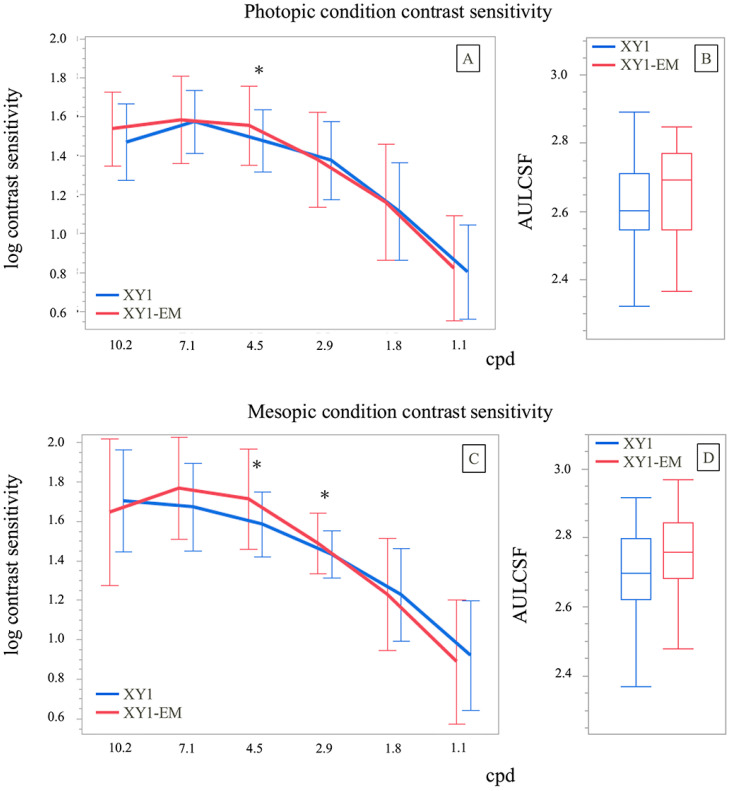
Postoperative contrast sensitivity. (**A**,**C**) Contrast sensitivity under mesopic and photopic conditions. (**B**,**D**) Area under the log contrast sensitivity function (AULCSF) under mesopic and photopic conditions. * *p* < 0.05.

Contrast sensitivity under photopic conditions was significantly different between groups only at 4.5 cpd (1.48 ± 0.16 vs. 1.55 ± 0.20; *p* = 0.04). Under mesopic conditions, the XY1-EM group demonstrated better contrast sensitivity at 2.9 cpd (1.59 ± 0.16 vs. 1.71 ± 0.25; *p* = 0.01) and 4.5 cpd (1.43 ± 0.12 vs. 1.49 ± 0.15; *p* = 0.02). The AULCSF showed no significant differences between groups under both photopic (*p* = 0.26) and mesopic (*p* = 0.20) conditions (Figure 4).

### 3.5. Patient-Reported Outcomes

The QOV questionnaire results showed no significant differences between groups for any parameter (all *p* > 0.05), indicating similar visual phenomena between the two lens types (Table 2).

**Figure 5:**
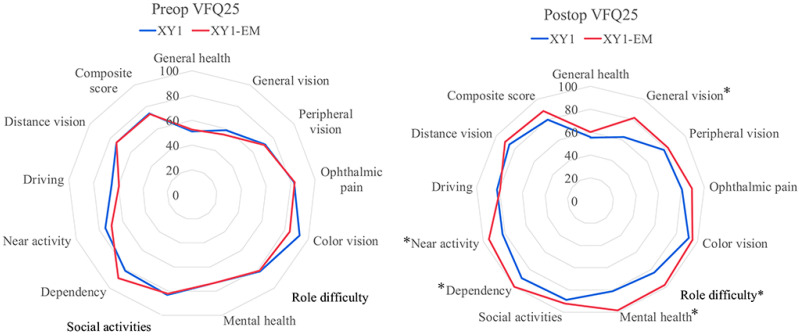
Postoperative VFQ-25 scores in the two study groups. * *p* < 0.05.

In the VFQ-25, no significant preoperative differences were found between groups. Postoperatively, the XY1-EM group demonstrated significantly higher scores in general vision (*p* = 0.01), role difficulty (*p* = 0.02), mental health (*p* < 0.001), dependency (*p* = 0.01), and near activity (*p* = 0.03) domains. Scores for general health, peripheral vision, ophthalmic pain, and driving were comparable between groups (Figure 5).

## 4. Discussion

This study compared the postoperative outcomes of standard monofocal IOLs (XY1) and enhanced monofocal IOLs (XY1-EM) in mini-monovision cataract surgery. The results demonstrated that the XY1-EM group achieved superior intermediate and near visual acuity, reduced spectacle dependence for intermediate distances, and significant improvements in vision-related quality of life compared to the XY1 group.

While the visual acuity at 5 m to 70 cm showed no significant differences between groups, the XY1-EM group exhibited significantly better visual acuity at near distances (50 cm to 30 cm). These findings suggest that mini-monovision surgery with the XY1-EM lens effectively improves intermediate-to-near vision, enhancing patients’ overall visual experiences. This improvement is likely attributable to the enhanced monofocal design, which extends the depth of focus at intermediate distances. Unlike the all-distance visual acuity assessed in this study, reports comparing defocus visual acuity under distance correction with the uncorrected distance visual acuity of emmetropic eyes implanted with the XY1-EM have shown approximately equivalent results [13].

Importantly, there were no significant differences in the QOV scores between groups, indicating that the XY1-EM does not increase visual side effects in patients any more than standard monofocal lenses do. This represents a significant advantage over multifocal IOLs, which often introduce visual disturbances such as glare, halos, and reduced contrast sensitivity.

The results of the VFQ-25 revealed significant improvements in the XY1-EM group for domains such as general vision, role difficulty, mental health, dependency, and near activity. These findings highlight the ability of the XY1-EM lens to provide functional vision across a broader range of daily tasks, thereby reducing spectacle dependence and enhancing overall quality of life.

Previous studies have established that monovision surgery with monofocal IOL implantation is associated with fewer visual disturbances and greater patient satisfaction compared to multifocal IOL implantation [14,15,16,17]. Our study is the first to demonstrate that mini-monovision cataract surgery with XY1-EM lens implantation extends the depth of focus for intermediate-to-near distances while maintaining low levels of visual side effects. These findings suggest that the XY1-EM lens offers a highly effective option for presbyopic correction and spectacle independence.

This study has several limitations. First, as a retrospective observational study, it is inherently more subject to bias than prospective randomized trials. Second, the sample size was relatively small, which may limit the generalizability of our findings. Prospective studies with larger populations are required to validate our results and evaluate long-term changes in visual performance. Furthermore, comparative studies with other enhanced monofocals, EDOF, and multifocal IOLs are necessary to establish the relative efficacy of the XY1-EM lens.

## 5. Conclusions

The XY1-EM enhanced monofocal IOL in mini-monovision configuration provides significantly better intermediate and near visual acuity compared to standard monofocal IOLs. Mini-monovision with XY1-EM significantly reduces spectacle dependence for intermediate distances without increasing visual side effects, and patients report higher quality of life scores in domains related to vision-dependent activities. This approach offers a valuable alternative for patients seeking improved QOV with reduced spectacle use following cataract surgery.

## Figures and Tables

**Table 1:** Characteristics of the study patients.

Characteristic	XY1 Mean ± SD (Min, Max)	XY1-EM Mean ± SD (Min, Max)	*p* Value
Eyes	66	48	
Female	19	13	0.55
Age (years)	72.06 ± 8.34 (62, 85)	71.33 ± 7.83 (49, 86)	0.24
IOL power (mm)	19.87 ± 4.18 (11.5, 27)	20.56 ± 2.80 (14.5, 27)	0.34
Axial length (mm)	24.07 ± 1.31 (21.44, 26.47)	23.95 ± 0.93 (21.32, 25.37)	0.84
ACD (mm)	3.04 ± 0.41 (1.68, 3.28)	3.09 ± 0.35 (1.72, 3.32)	0.66
Kf (mm)	7.72 ± 0.33 (7.25, 8.15)	7.73 ± 0.24 (7.16, 8.32)	0.72
Ks (mm)	7.54 ± 0.33 (7.06, 7.96)	7.66 ± 0.24 (7.07, 8.17)	0.02 *
CDE	4.25 ± 2.08 (2.39, 11.07)	4.05 ± 2.31 (0, 11.28)	0.45
CDVA (logMAR)	−0.12 ± 0.09 (−0.18, 0.22)	−0.1 ± 0.10 (−0.18, 0.05)	0.29

**Abbreviations:** XY1, Vivinex monofocal intraocular lens; XY1-EM, Vivinex Impress enhanced monofocal intraocular lens; IOL, intraocular lens; Kf, flat keratometry; Ks, steep keratometry; ACD, anterior chamber depth; CDE, cumulative dissipated energy; CDVA, corrected distance visual acuity; SD, standard deviation. * A *p* value < 0.05 was considered statistically significant.

**Table 2:** Quality of vision questionnaire results.

	XY1		XY1-EM		
	Mean	SD	Mean	SD	*p*
Glare frequency	0.05	0.22	0.10	0.30	0.42
Glare severity	0.15	0.49	0.15	0.48	0.98
Glare bothersome	0.05	0.22	0.05	0.22	0.99
Halo frequency	0.10	0.31	0.20	0.61	0.92
Halo severity	0.15	0.49	0.15	0.48	0.98
Halo bothersome	0.00	0.00	0.05	0.22	0.17
Starburst frequency	0.46	0.82	0.50	0.99	0.74
Starburst severity	0.56	0.88	0.45	0.88	0.55
Starburst bothersome	0.15	0.37	0.10	0.30	0.48
Hazy frequency	0.08	0.48	0.20	0.52	0.06
Hazy severity	0.08	0.48	0.20	0.52	0.06
Hazy bothersome	0.08	0.48	0.05	0.22	0.60
Blurred frequency	0.08	0.48	0.20	0.69	0.19
Blurred severity	0.08	0.48	0.15	0.48	0.20
Blurred bothersome	0.08	0.48	0.15	0.48	0.20
Distortion frequency	0.00	0.00	0.00	0.00	1.00
Distortion severity	0.00	0.00	0.00	0.00	1.00
Distortion bothersome	0.00	0.00	0.00	0.00	1.00
Multiple image frequency	0.18	0.56	0.05	0.22	0.22
Multiple image severity	0.18	0.56	0.10	0.44	0.25
Multiple image bothersome	0.13	0.52	0.05	0.22	0.62
Fluctuation frequency	0.05	0.22	0.15	0.48	0.40
Fluctuation severity	0.05	0.22	0.15	0.48	0.40
Fluctuation bothersome	0.05	0.22	0.05	0.22	0.99
Focusing difficulties frequency	0.44	0.79	0.20	0.41	0.27
Focusing difficulties severity	0.44	0.79	0.20	0.41	0.27
Focusing difficulties bothersome	0.23	0.67	0.15	0.36	0.90
Distance frequency	0.00	0.00	0.05	0.22	0.17
Distance severity	0.00	0.00	0.05	0.22	0.17
Distance bothersome	0.00	0.00	0.00	0.00	1.00

No statistically significant differences were observed between the XY1 and XY1-EM groups in any of the QOV questionnaire items (all *p* > 0.05). **Abbreviations:** XY1, Vivinex monofocal intraocular lens; XY1-EM, Vivinex Impress enhanced monofocal intraocular lens; SD, standard deviation.

## Data Availability

Data supporting the results of this study are stored in the Tsukazaki Hospital archive. The datasets supporting the conclusions of this article are available from the corresponding author upon reasonable request.

## Abbreviations

The following abbreviations are used in this manuscript:

ACD	Anterior chamber depth
AULCSF	Area under the log contrast sensitivity function
CDE	Cumulative dissipated energy
CDVA	Corrected distance visual acuity
EDOF	Extended depth-of-focus
IOL	Intraocular lens
QOV	Quality of vision
UCVA	Uncorrected visual acuity
